# Multi-Parameter MR Radiomics Based Model to Predict 5-Year Progression-Free Survival in Endometrial Cancer

**DOI:** 10.3389/fonc.2022.813069

**Published:** 2022-03-31

**Authors:** Defeng Liu, Linsha Yang, Dan Du, Tao Zheng, Lanxiang Liu, Zhanqiu Wang, Juan Du, Yanchao Dong, Huiling Yi, Yujie Cui

**Affiliations:** ^1^ Medical Imaging Center, First Hospital of Qinhuangdao, Qinhuangdao, China; ^2^ Department of Intervention, First Hospital of Qinhuangdao, Qinhuangdao, China

**Keywords:** endometrial cancer, progression-free survival, radiomics, magnetic resonance imaging, nomogram

## Abstract

**Background:**

Relapse is the major cause of mortality in patients with resected endometrial cancer (EC). There is an urgent need for a feasible method to identify patients with high risk of relapse.

**Purpose:**

To develop a multi-parameter magnetic resonance imaging (MRI) radiomics-based nomogram model to predict 5-year progression-free survival (PFS) in EC.

**Methods:**

For this retrospective study, 202 patients with EC followed up for at least 5 years after hysterectomy. A radiomics signature was extracted from T2-weighted imaging (T2WI), apparent diffusion coefficient (ADC) and a dynamic contrast-enhanced three-dimensional volumetric interpolated breath-hold examination (3D-VIBE). The radiomics score (RS) was calculated based on the least absolute shrinkage and selection operator (LASSO) regression. We have developed a radiomics based nomogram model (Model^N^) incorporating the RS and clinical and conventional MR (cMR) risk factors. The performance was evaluated by the receiver operating characteristic curve (ROC), calibration curve and decision curve analysis (DCA).

**Results:**

The Model^N^ demonstrated a good calibration and satisfactory discrimination, with a mean area under the curve (AUC) of 0.840 and 0.958 in the training and test cohorts, respectively. In comparison with clinical prediction model (Model^C^), the discrimination ability of Model^N^ showed an improvement with P < 0.001 for the training cohort and P=0.032 for the test cohort. Compared to the radiomics prediction model (Model^R^), Model^N^ discrimination ability showed an improvement for the training cohort with P = 0.021, with no statistically significant difference in the test cohort (P = 0.106). Calibration curves suggested a good fit for probability (Hosmer–Lemeshow test, P = 0.610 and P = 0.956 for the training and test cohorts, respectively).

**Conclusion:**

This multi-parameter nomogram model incorporating clinical and cMR findings is a valid method to predict 5-year PFS in patients with EC.

## Introduction

Endometrial cancer (EC) is one of the three most common malignancies of the female reproductive tract (1). Many clinical studies have shown that deciding whether to conduct radiotherapy or chemotherapy according to the risk of tumors can not only avoid unnecessary pain and economic burden brought by overtreatment of early-stage patients, but also avoid undertreatment of high-risk tumors, delay recurrence and improve the quality of life (2–4). Previous studies have proposed predicting the myometrial invasion and clinical outcome of EC by combining clinical and pathological indicators (5, 6). Tumor size, myometrial invasion, lymph vascular space invasion (LVSI) and other parameters obtained by postoperative tumor pathology can certainly be used to evaluate the EC prognosis. However, if we can use accurate, non-invasive methods to determine the risk and prognosis before surgery, it is beneficial to select more reasonable treatment strategies improve the progression-free and overall survival. Previous studies have found that EC prognosis is not only related to these pathological features, but also to the patient’s age, BMI and other clinical indicators (7, 8). Therefore, making full use of these preoperative indicators is instrumental to a more accurate prognosis prediction. Furthermore, postoperative pathological examinations are very invasive but with appropriate preoperative predictive methods many unnecessary surgeries could be avoided.

Previous studies have found that preoperative staging, prognosis, and survival of EC can be predicted by using clinical standard magnetic resonance imaging (MR) sequences to assess the deep myometrial invasion, tumor volume or maximum diameter, and lymph node invasion (9–11). T1-weighted imaging (T1WI) and T2-weighted imaging (T2WI), the most commonly used modalities, are mainly used to evaluate tumor nature and prognosis by observing morphological characteristics. However, their accuracy is limited by visual resolution and the observer diagnostic ability (10). Although functional imaging methods such as quantitative diffusion and perfusion MRI can help us judge the tumor nature, these advanced imaging methods have high requirements on the imaging equipment and post-processing software, which may limit their accessibility (12). Radiomics represent a set of tools extracting quantitative features from medical images evaluating tumor characteristics such as heterogeneity (13). Data mining through radiomics allows researchers to explore the tumor heterogeneity, which is closely related to tumor aggressiveness and prognosis (14, 15). Previous studies have reported radiomics feasibility in predicting the histologic grade of endometrial carcinoma, lymph node metastasis or LVSI, and deep myometrial invasion (DMI) (16–18). However, the correlation between radiomics parameters and the EC patient survival is still unknown. Therefore, this study aimed to develop a multi-parameter MRI radiomics-based nomogram model to predict 5-year progression-free survival (PFS) in EC. In order to assess what our model achieved using this full sample set, and that it was not biased by the inclusion of various stages and grades of EC, we also carried out a sensitivity analysis focused on the different stages and grades.

## Materials and Methods

### Patients

**Figure 1:**
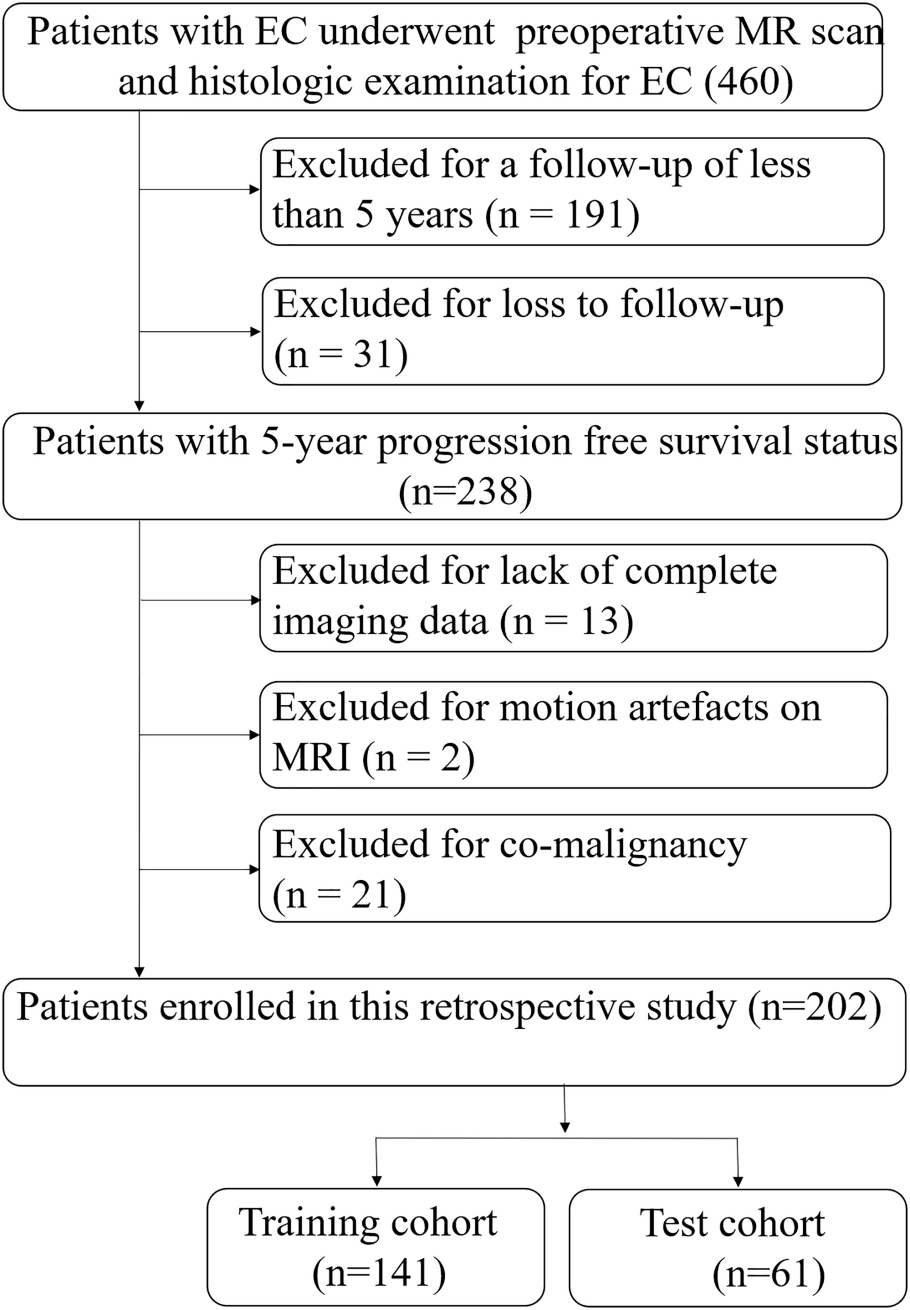
Recruitment pathway for patients in this study. EC, endometrial cancer.

This retrospective study was approved by our institution’s ethics committee. Informed consent was waived because analysis was performed on anonymized images and clinical data. A total of 460 patients with endometrial cancer confirmed by postoperative histopathology in our hospital from January 2011 to January 2016 were successively identified in the database. Inclusion criteria: (1) All patients underwent hysterectomy with bilateral salpingo-oophorectomy and were pathologically confirmed to be endometrial carcinoma, regardless of whether they had received radiotherapy or chemotherapy after surgery. (2) MR was performed within two weeks before surgery. Exclusion criteria: (1) No lesion that could be accurately identified in MR images or the maximum diameter of the lesion was less than 1 cm. (2) Lack of complete imaging data. (3) There are obvious artifacts in the image, which affect the observation. (4) Patients with co-malignancies. (5) Patients with further oncological diseases. (6) Follow-up less than 5 years or lost. 202 patients were enrolled in the study, and patients were randomly assigned to two separate cohorts, namely the training cohort (n=141) and the test cohort (n=61), in a 0.7:0.3 ratio (**Figure 1**).

### Clinical Data

Clinical indicators collected preoperatively included patients’ age, Body Mass Index (BMI), hypertension and diabetes, Carbohydrate Antigen 125 (CA125) and Human Epididymis Protein 4 (HE4) levels, which were obtained from our Hospital Information System (HIS). BMI = weight/height2 (kg/m2). Hypertension is defined as a systolic blood pressure of 140 mmHg or greater and/or a diastolic blood pressure of 90 mmHg or greater. Diabetes is defined as fasting blood glucose greater than or equal to 7.0 mmol/L and/or postprandial blood glucose greater than or equal to 11.1 mmol/L. The CA125 and HE 4 level was detected by chemiluminescence microparticle immunoassay (Cobas 8000 E602; Roche Holding AG).

### Follow-Up

Progression was defined as local recurrence progression in the pelvis or new metastases in the abdomen or at distant sites, including nodal, peritoneal, or visceral metastases. All patients were consistently followed up every 3 to 6 months after surgery based on the thoracic, abdominal and pelvic CT or abdominal and pelvic MR imaging to determine if there is progression. The images were independently evaluated by two radiologists, neither of whom was aware of the EC stage or subtype. If the two radiologists cannot agree on the assessment of the metastasis status, another more experienced radiologist will conduct the assessment until a consensus is reached.

PFS is defined as the time when a patient receives surgical treatment until disease progression is observed or death from any cause occurs. Patients who relapsed or died within 5 years were assigned to the high-risk (HR) group, while those who did not relapse were assigned to the low-risk (LR) group.

### MRI Scan

Images were collected for all patients using a 1.5 T MR scanner (Avanto, Siemens) equipped with an 8-channel body coil. The scanning area ranged from the anteroom-superior iliac spine to the symphysis pubis. The scanning sequence included the coronal, sagittal, and axial oblique fat-saturation (fs) T2WI; axial oblique DWI and axial oblique three-dimensional volumetric interpolated breath-hold examination (3D-VIBE). DWI was acquired by echo-planar imaging (b-value = 0, 800 s/mm^2^). After DWI scanning, the workstation automatically calculates and generates ADC map. The specific MRI parameters are shown in **Table 1**. When 3D-VIBE sequence was used to obtain DCEI, patients were instructed to hold their breath at the end of expiratory breath. A high pressure syringe (Spectris MR injection system, Medrad Inc.) was used to administer gadolinium diethylenetriamine penta-acetic acid (Bayer Healthcare Pharmaceuticals) through the cubital vein at a rate of 2 mL/s. The dosage of Gd-DTPA was 0.1 mmol/kg. Images of arterial phase, venous phase and delay phase were obtained by scanning at 25 s, 60 s and 180 s after administration.

**Table 1:** MRI Scanning Protocols.

Sequences	Plane	FS	TR/TE (ms)	FA (deg)	Slice thickness/Interslice gap (mm)	Matrix	FOV (mm)	Pixel size (mm)
T2TSE	SAG	Yes	4340/92	150	4/0.4	320×256	280×224	0.9×0.9
T2TSE	COR	Yes	4340/92	150	4/0.4	320×256	280×224	0.9×0.9
T2TSE	AO	Yes	4340/92	150	4/0.4	320×256	280×224	0.9×0.9
DWI	AO	Yes	7000/80	90	4/0.4	256×205	280×224	1.1×1.1
VIBE	AO	Yes	4.44/2.16	10	4/0	320×256	280×224	0.9×0.9

AO, axial oblique slice orientation; COR, coronal slice orientation; Deg, degrees; DWI: diffusion weighted imaging; FA, flip angle; FOV, field of view; SAG, sagittal slice orientation; TE, time echo; TR, repetition time; TSE, turbo spin echo; VIBE, volumetric interpolated breath-hold examination.

### MRI Evaluation

Additionally, all EC scans were independently evaluated by two radiologists with more than 10 years of experience in pelvic MRI diagnosis. The collected MR indicators included positive/negative DMI, maximum tumor diameter, positive/negative pelvic lymph nodes (PLN), and positive/negative abdominal para-aortic lymph nodes (PALN). A lymph node with a short diameter of ≥1cm or with circular enhancement with central necrosis on enhanced scan is considered positive (19). After the evaluation, the intra-class correlation coefficient (ICC) and Kappa value of each index reported by the two radiologists were calculated. If the two indexes were greater than 0.75, the parameters were considered to be stable.

### Tumor Image Segmentation and Radiomics Parameter Extraction

**Figure 2:**
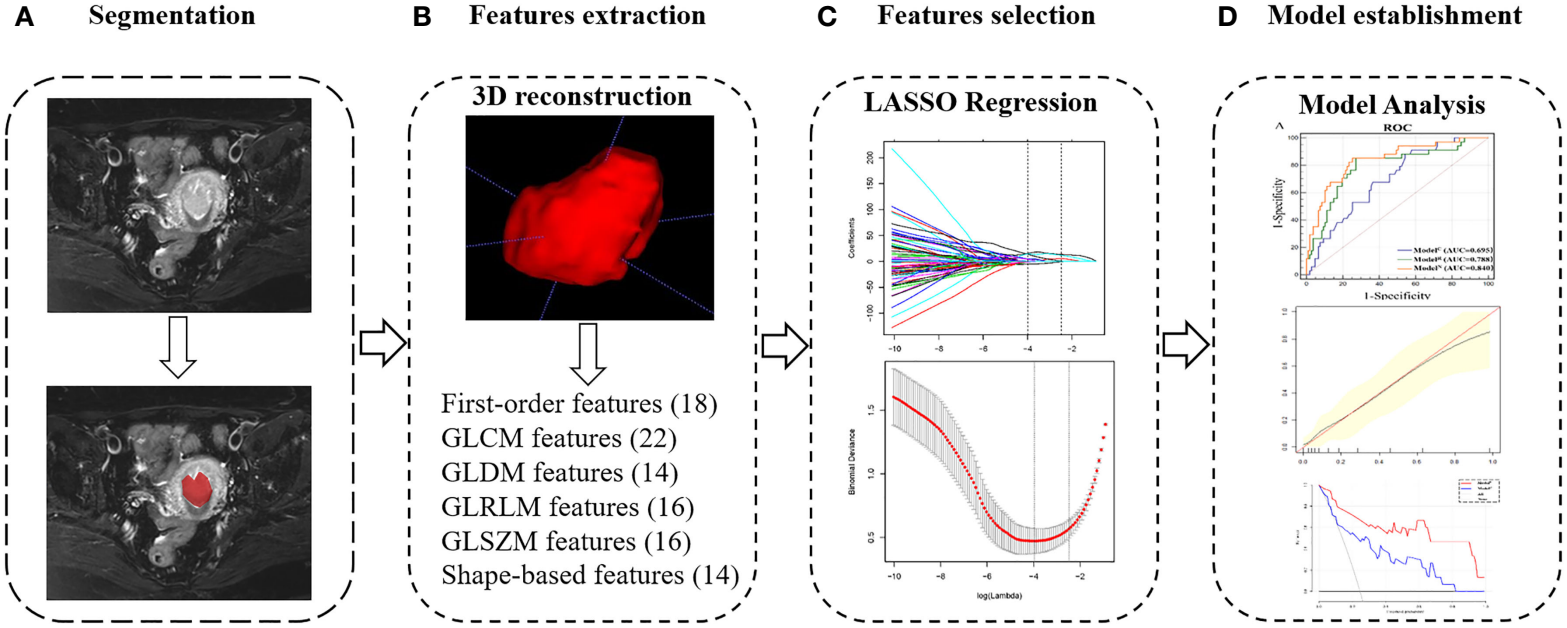
Radiomics workflow of model construction. **(A)** MR images segmentation. The tumor was segmented manually on the axial T2-weighted images. **(B)** Texture features extraction. After 3D reconstruction, a total of 100 texture parameters of 6 types were extracted from each set of images. **(C)** Texture features selection. After the parameters were normalized and dimensionality reduced, the characteristic parameters were selected and classified by LASSO regression. **(D)** Model establishment. Combined with two clinical indicators of location and size, nomogram was developed to establish a preoperative evaluation model, and it was evaluated according to receiver-operating characteristic, calibration and decision curves.

First, the axial diffusion fs-T2WI, DWI and 3D-VIBE images were downloaded from the Picture Archiving and Communication System (PACS). Axial-T2WI was used as a reference image, and axial-ADC, axial-3D-VIBE are registered to T2WI using Statistical Parametric Mapping software 12 (SPM12; University College London). Subsequently, the two radiologists mentioned above performed a layer-by-layer manual delineation of the volume of interest (VOI) on T2WI for all patients, focusing on covering the entire tumor. VOIs and tumor images, including T2WI, ADC and arterial, venous and delayed 3D-VIBE are imported one by one into the PyRadiomics toolkit version 3.0. The ICC of each parameter extracted by the radiologists was calculated, and the ICC>0.75 parameter was included. **Figure 2** and **Supplementary Methods S1** illustrate the process of VOI delineation, parameter extraction, and modelling in a patient with EC.

### Feature Selection and Radiomics Signature Construction

The radiomics data is normalized and pre-processed using FAE software (FAE, https://github.com/salan668/FAE, version 0.3.6). “Normalise to unit” was used to normalize the data in order to reduce large differences in the values of the different radiomics features. Pearson’s correlation coefficients (PCCs) were calculated for pre-processing. When the PCC is larger than the threshold value, one of the radiomics feature is removed randomly. See the **Supplementary Methods S2** for specific methods and calculation formulas. The Using X&Y software (X&Y Solutions, Inc.), the parameters related to PFS status were selected using the Least Absolute Shrinkage and Selection Operator (LASSO) regression method in the training cohort (**Supplementary Method S3**). The individualised radiomics based nomogram model, incorporating the radiomics signature and independent clinical risk factors, was constructed using the logistic regression.

### Assessment and Validation of Model Performance

The area under the curve obtained by ROC analysis was used to evaluate the differentiating ability of the model (20). Calibration curves were used to assess the predictive power of the model, and the actual classification and the Hosmer–Lemeshow test was performed to assess the goodness-of-fit (21). The clinical efficacy of the model was evaluated by using the net benefits with different threshold probabilities obtained from the decision curve analysis (DCA) in the test cohort (22).

We also conducted sensitivity analysis of for our prediction Model^N^ to judge the diagnostic efficiency of the model between different pathological grades and stages. The FIGO staging criteria revised in 2009 for EC were used for histological diagnosis, grading, and pathological staging (23).

Pathological grading subgroup: All patients (including the training and test cohorts) were divided into two subgroups according to pathological grades. G1 and G2 endometrioid adenocarcinoma were classified as low-grade subgroup, and G3 or non-endometrial carcinoma (clear cell adenocarcinoma, serous adenocarcinoma, etc.) was classified as high grade subgroup (24).

Pathological staging subgroup: All patients (including training and test cohorts) were divided into two subgroups according to their pathological stages. Stage I and II patients were defined as low stage subgroup, while III and IV patients were defined as high stage subgroup.

### Statistical Analysis

All statistical analyses were conducted using X&Y software based on R software. Univariable and multivariable logistic regression analyses were performed to identify the independent clinical risk factors associated with 5-year PFS. The candidate factors for univariable analysis were age, BMI, HE4, CA125, hypertension, diabetes, maximum diameter, DMI, PALN, PLN. Beta value, odds ratio and their 95% confidence interval (CI) were calculated. The variables with a P-value <0.10 in the univariable and multivariable analysis were selected as independent risk factors. A two-tailed P-value< 0.05 was considered statistically significant.

## Results

### Clinical and MR Indicators

A total of 202 patients were eventually enrolled and analysed based on the inclusion and exclusion criteria. According to their 5-year PFS status, there were 49 cases in the high-risk group and 153 cases in the low-risk group. The ICCs of clinical and MR indicators were greater than 0.75, indicating a good agreement between the two measurements. Sample sizes, baseline clinical characteristics and pathological characteristics of the two groups are shown in **Tables 2**, **3**. Subsequently, univariate and multivariate analyses showed that there were significant differences in age, BMI, HE4, maximum diameter, and PALN between the two groups (all P<0.05, **Table 4**). These factors could be independent clinical risk factors for preoperative evaluation of 5-year survival status of EC patients.

**Table 2:** Patient characteristics in the training and test cohorts.

Characteristics	Training cohort (*n* = 141)			Test cohort (*n* = 61)		*P-*value
	LR (*n* = 107)	HR (*n* = 34)	*P-*value	LR (*n* = 46)	HR (*n* = 15)	
Age (years)			**0.021**			0.729
Mean ± SD	51.1 ± 12.8	57.3 ± 15.0		53.8 ± 12.9	61.1 ± 10.9	
Range	30.0-88.0	35.0-90.0		30.0-94.0	45.0-75.0	
BMI			**0.007**			**0.009**
Mean ± SD	26.4 ± 6.2	29.8 ± 6.6		26.5 ± 6.9	30.3 ± 6.2	
Range	19.8-39.5	21.1-40.1		19.1-38.5	21.8-38.3	
CA125			0.118			0.586
Mean ± SD	91.7 ± 31.4	327.4 ± 272.9		93.3 ± 26.7	97.2 ± 32.5	
Range	16.0-162.0	21.0-160.0		32.0-141.0	39.0-154.0	
HE4			**0.043**			**0.041**
Mean ± SD	112.7 ± 43.5	130 ± 40.3		111.8 ± 41.6	139.7 ± 54.1	
Range	23.0-264.0	58.0-243.0		12.0-257.0	41.0-207.0	
Hypertension			0.285			0.493
No	68 (63.6%)	25 (73.5%)		32 (69.6%)	9 (60.0%)	
Yes	39 (36.4%)	9 (26.5%)		14 (30.4%)	6 (40.0%)	
Diabetes			0.703			0.076
No	73 (68.2%)	22 (64.7%)		33 (71.7%)	7 (46.7%)	
Yes	34 (31.8%)	12 (35.3%)		13 (28.3%)	8 (53.3%)	
Maximum Diameter			**0.002**			0.081
Mean ± SD	4.1 ± 1.0	4.8 ± 1.0		4.0 ± 1.1	4.6 ± 1.1	
Range	2.1-6.2	3.2-6.5		2.3-6.1	2.5-5.7	
DMI			0.363			0.597
No	44 (41.1%)	17 (50.0%)		22 (47.8%)	6 (40.0%)	
Yes	63 (58.9%)	17 (50.0%)		24 (52.2%)	9 (60.0%)	
PLN			0.132			0.929
No	66 (61.7%)	16 (47.1%)		27 (58.7%)	9 (60.0%)	
Yes	41 (38.3%)	18 (52.9%)		19 (41.3%)	6 (40.0%)	
PALN			**0.019**			0.076
No	87 (81.3%)	21 (61.8%)		40 (87.0%)	10 (66.7%)	
Yes	20 (18.7%)	13 (38.2%)		6 (13.0%)	5 (33.3%)	

LR, low-risk group; HR, high-risk group; BMI, body mass index; presence of hypertension and diabetes; CA125, carbohydrate antigen 125; HE4, human epididymis protein 4; HBP, high blood pressure; DMI, deep myometrial invasion; PLN, pelvic lymph nodes; PALN, para-aortic lymph nodes. P value was derived from the student-t or chi-square test. Bold type indicates statistically significant difference.

**Table 3:** Pathological characteristics of the patients in our study.

Characteristics	LR (*n* = 153)	HR (*n* = 49)	*P*-value
Pathological staging, n (%)			**0.0004**
pI	53 (34.6%)	15 (30.6%)	
pII	74 (48.4%)	16 (32.7%)	
pIII	20 (13.1%)	14 (28.6%)	
pIV	6 (3.9%)	4 (8.1%)	
Histological grade, n (%)			**0.0232**
G1	71 (46.4%)	13 (26.5%)	
G2 G3 and non-endometrial carcinoma	48 (31.4%)	17 (34.7%)	
	34 (22.2%)	19 (38.8%)	

LR, low-risk group; HR, high-risk group. Bold type indicates statistically significant difference.

**Table 4:** Preoperative clinical risk factors for 5-year PFS in patients with EC.

Variable	Univariable analysis		Multivariable analysis	
	OR (95% CI)	*P*-value	OR (95% CI)	*P*-value
Age (years)	1.037 (1.012, 1.062)	**0.004**	1.035 (1.007, 1.064)	**0.013**
BMI	1.085 (1.032, 1.140)	**0.001**	1.084 (1.025, 1.146)	**0.005**
CA125	1.000 (1.000, 1.000)	0.114		
HE4	1.010 (1.003, 1.018)	**0.006**	1.011 (1.003, 1.020)	**0.010**
Hypertension		0.604		
No	1.000			
Yes	0.832 (0.416, 1.664)			
Diabetes		0.193		
No	1.000			
Yes	1.555 (0.800, 3.025)			
Maximum Diameter	1.934 (1.367, 2.737)	**<0.001**	2.000 (1.362, 2.936)	**<0.001**
DMI		0.641		
No	1.000			
Yes	0.858 (0.450, 1.636)			
PLN		0.229		
No	1.000			
Yes	1.488 (0.779, 2.843)			
PALN		**0.004**		**0.023**
No	1.000		1.000	
Yes	2.836 (1.383, 5.814)		2.590 (1.140, 5.885)	

BMI, body mass index; presence of hypertension and diabetes; CA125, carbohydrate antigen 125; HE4, human epididymis protein 4; HBP, high blood pressure; DMI, deep myometrial invasion; PLN, pelvic lymph nodes; PALN, para-aortic lymph nodes. Bold type indicates statistically significant difference.

### Evaluation of Diagnostic Efficacy of Clinical Indicators

**Figure 3:**
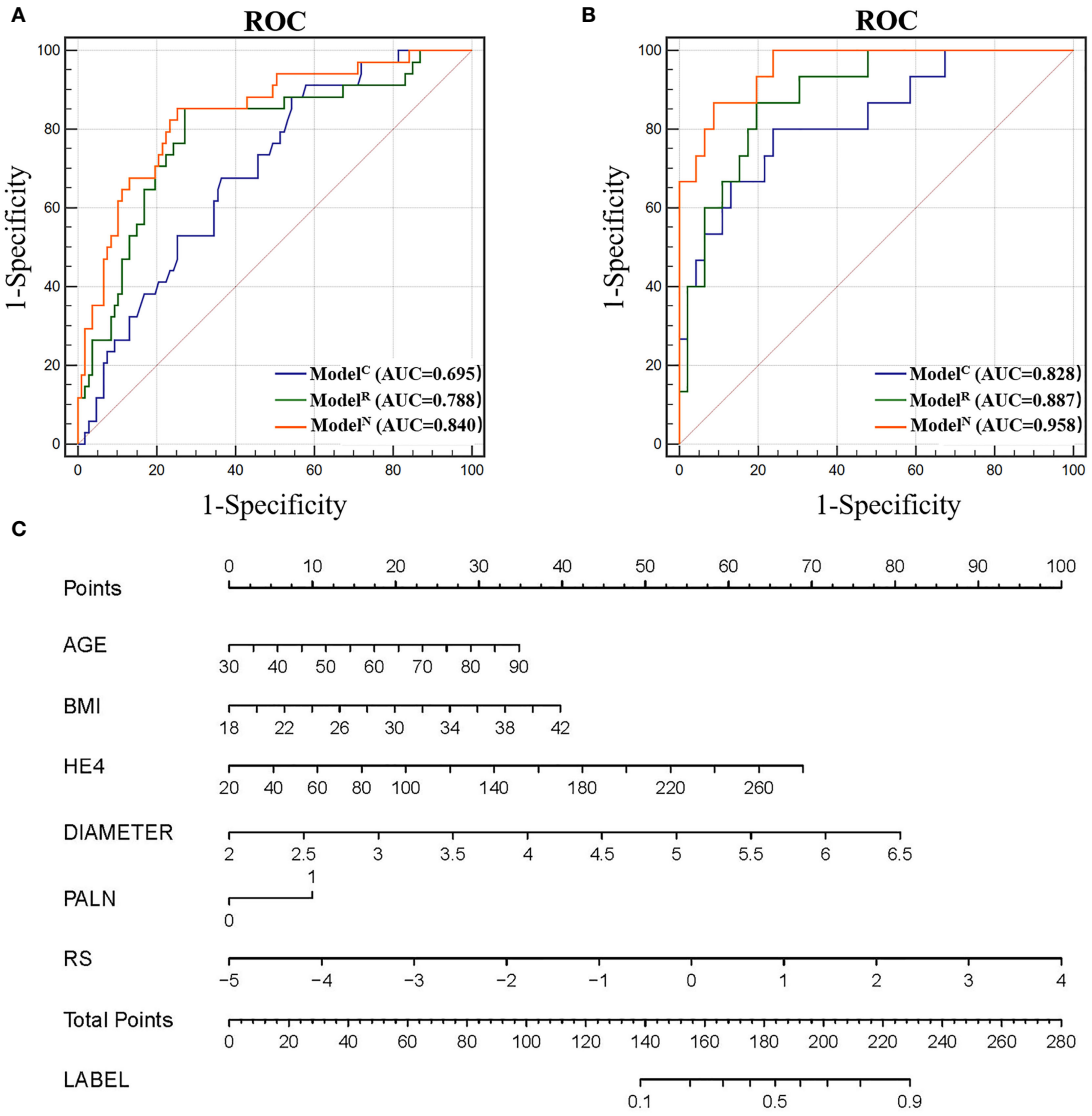
**(A)** Receiver operating characteristic (ROC) of different models in the training cohort. **(B)** ROC of different models in the test cohort. **(C)** Nomogram for predicting risk classification of EC. The nomogram was built in the training cohort with the Radscore, BMI and CA125. The probability of each predictor can be converted into scores according to the first scale points at the top of the nomogram. After adding up the scores of these predictors in total points, the corresponding prediction probability at the bottom of the nomogram is the malignancy of the tumor.

Four independent clinical risk factors including age, BMI, HE4, maximum diameter and PALN were combined with logistic regression to construct a clinical prediction model (Model^C^) and develop a ROC curve to evaluate the preoperative prediction ability of the model for tumors of two different risk grades. The AUCs were 0.695 [95% confidence interval (CI), 0.612-0.770] and 0.828 (95% CI, 0.709–0.912) for the training and test cohorts, respectively (**Figure 3**).

### Radiomics Score (RS) and the Diagnostic Efficacy for Our Radiomics Model

A total of 4 parameters with a non-zero coefficient are selected by LASSO regression, namely, ADC entropy, ADC Kurtosis, T2 Kurtosis, and Arterial HGLRE. RS values for each patient were calculated based on their respective coefficients in regression equation. Equation is as follows: RS=0.19213+1.97154* ADC entropy+37.45352* ADC kurtosis+13.73094* T2 kurtosis+10.94133* Arterial HGLRE.

The RS for each patient was calculated to build radiomics model (Model^R^) to predict 5-PFS. The AUCs for Model^R^ were 0.788 [95% CI, 0.712-0.853] and 0.887 (95% CI, 0.780–0.954) for the training and test cohorts, respectively (**Figure 3**). There was no statistically significant difference between the AUC of Model^R^ and Model^C^ (P=0.167 and 0.493 for the training and test cohort, respectively).

### Radiomics Based Nomogram Model (Model^N^) Establishment and Performance

**Figure 4:**
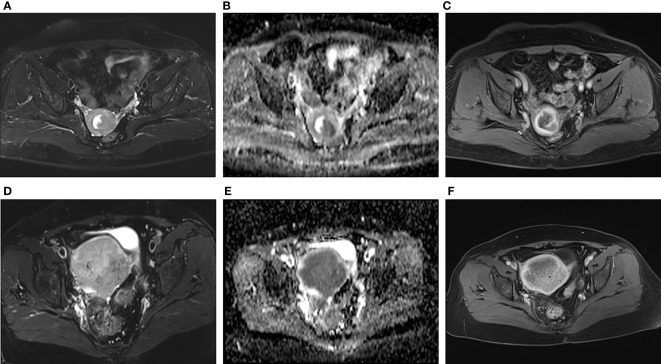
Representative images of two patients with different survival outcomes. **(A–C)** axial oblique T2WI, ADC map and arterial phase images of a 55-year-old woman with low-risk EC. The nomogram model (Model^N^) score was -1.37. Using the nomogram, the estimated probability of relapse or death within 5 years was 17%. The tumor did not recur during the 5-year observation period. **(D–F)** axial oblique T2WI, ADC map and arterial phase images of a 75-year-old woman with high-risk EC. The Model^N^ score was 2.92. Using the nomogram, the estimated probability of relapse or death within 5 years was 95%. The tumor relapsed 6 months after surgery.

The addition of radiomics parameters can improve the discriminative ability of Model^C^. The nomogram achieved excellent performance in predicting risk grading, with AUC of 0.840 (95% CI: 0.769–0.896) in the training and 0.958 (95% CI: 0.873–0.993) in the test cohort. The predictive ability of the nomogram was better than that of the Model^C^ in the training cohort (P<0.001) and test cohort (P=0.032). The predictive ability of the nomogram was also better than that of the Model^R^ in the training cohort (P=0.021). However, in the test cohort, there was no statistically significant difference between the AUC of Model^N^ and Model^R^ (P = 0.106). In **Figure 4**, we include two representative MRI images illustrating visually striking differences in tumor heterogeneity between a patient who survived 26 months and another who succumbed at 8 months. T2WI, ADC and arterial-phased 3D-VIBE images of two representative cases with their Model^N^ score and predicted 5-year PFS are shown in **Figure 4**.

**Figure 5:**
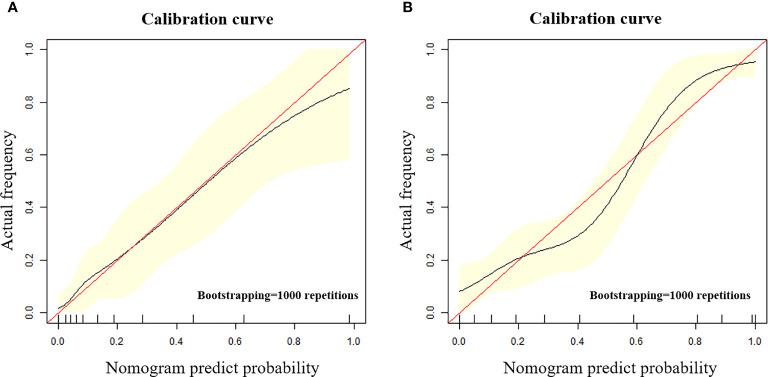
**(A, B)** The calibration curve in the training cohort **(A)** and test cohort **(B)**. The calibration curve depicted the agreement between the predicted risk classification score and the actual results confirmed by examination. The red line represents an ideal prediction, and the black line represents the predictive performance. The closer the fit of the black line to the ideal line, the better the prediction.

**Figure 6:**
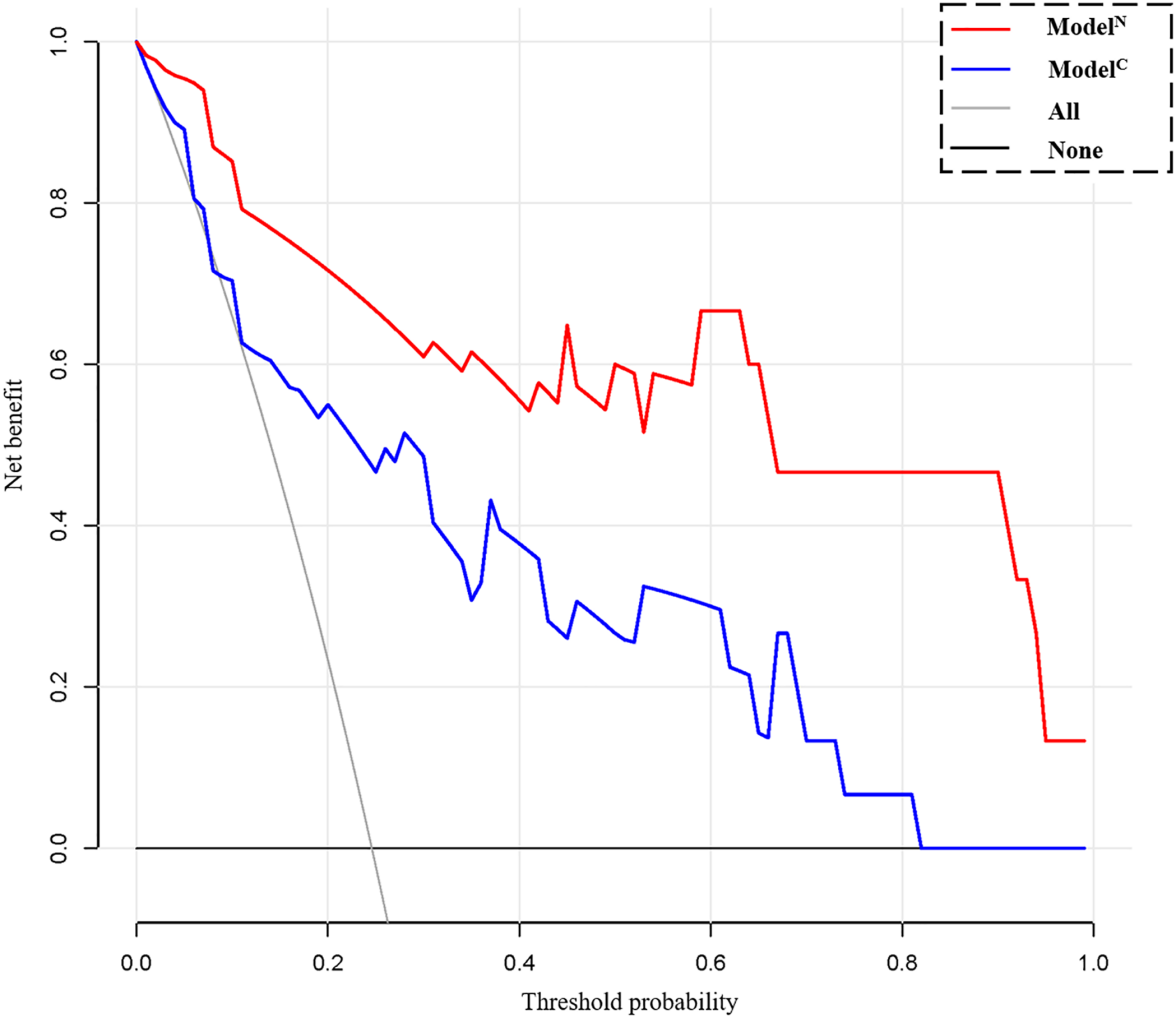
Decision curve analysis (DCA) for the clinical model (Model^C^) and nomogram model (Model^N^). The decision curve showed that using a clinical- radiomics combined model to predict 5-year progression-free survival (PFS) of endometrial cancer would be more beneficial than the clinical model.

The calibration curve shows that the predicted value of the model is in good agreement with the actual value (P = 0.610 and P = 0.956 for the training and test cohorts, respectively). We calculated the risk scores for all patients in the training set and the test set to visually display the prediction ability of the model (**Figure 5**). The DCA indicates that the clinical application of Model^N^ has a better performance than that of Model^C^, which also added more benefit than assuming that all cases are positive (high-risk EC) or negative (high-risk EC) (**Figure 6**).

In the subgroup analysis, Model^N^ had a good differential diagnostic capability in all subgroups divided according to different criteria and there was no significant difference between subgroups. The AUC of the low- and high-grade subgroups were 0.871 and 0.926. The AUC of the low stage and high stage subgroups were 0.873 and 0.831. There was also no significant difference between the subgroups of different grade and stage (**Figure S1**).

## Discussion

In this study, a radiomics model based on multi-parameter MRI has been established to predict 5-year PFS in EC patients. The model combined the radiomics parameters obtained by preoperative MR examination with those easily obtained preoperatively to provide predictive information for long-term prognosis. Compared with clinical and radiomics models, this comprehensive model provides better discrimination ability, enabling clinicians to grade tumor risk preoperatively, which can be used to guide treatment decisions. The discriminative ability of nomogram model discrimination ability was also demonstrated in subgroup analysis.

Four radiomics parameters (ADC entropy, ADC Kurtosis, T2 Kurtosis, Arterial HGLRE) are selected to calculate the RS in this study. Several previous studies have used radiomics-based models to predict a prognosis in a variety of tumors (25–27). For example, recent studies have shown that RS-based models can predict EC lymph node metastasis and LVSI (16, 28). A previous CT study has found that a high tumor entropy independently predicted deep myometrial invasion (odds ratio [OR] 3.7, p=0.008) and cervical stroma invasion (OR 3.9, p=0.02) (29). In addition, a high tumor kurtosis tends to independently predict a reduced recurrence- and PFS (HR 1.1, p=0.06) (29). Another MR study also found that MR was a sensitive indicator for PFS. High kurtosis in T1 c images predicted a reduced recurrence- and progression-free survival (hazard ratio [HR] 1.5, P < 0.001) after adjusting for MRI-measured tumor volume and histological risk at biopsy (30). High tumor entropy in apparent diffusion coefficient (ADC) maps independently predicted deep myometrial invasion (odds ratio [OR] 3.2, P < 0.001) (30). It is not difficult to find that the parameters screened in the above studies are similar to the radiomics parameters in this study.

The parameters screened in this study also overlapped with other tumor prognostic parameters. A previous study has shown that a radiomics model can more accurately predict 3-year and 5-year PFS for advanced nasopharyngeal carcinoma than a clinical model based on TNM stage alone (31). A previous breast cancer study found that entropy can be used as a predictor of benign, malignant and risk assessment of tumors, with an AUC of 0.8 and a sensitivity of 95% when applied alone (32). A study found that Kurtosis combined with several clinical and other texture parameters could predict eight-year event-free survival (EFS) in Luminal Non-Metastatic Breast Valencia (33). In addition to adenocarcinoma, another study on anal squamous cell carcinoma also found that Entropy and Joint Energy can be independent risk factors for predicting tumor recurrence rate (34). Therefore, the radiomics indicators screened in this study are not only reproducible in the evaluation of multiple biological characteristics and prognosis of endometrial cancer, but also seem to be similar in other tumors.

Previous studies generally report that the older the onset age, the higher the risk of endometrial cancer recurrence and death (7). This may be due to the fact that elderly patients are more prone to high-grade or specific histological types of EC and to various complication (35). This study also found that the higher the BMI for our EC patients, the more likely they were to relapse, which may be related to the increase of oestrogen level caused by obesity (8). In addition, HE4 has recently been identified as a potential biomarker for endometrial cancer with higher sensitivity than CA125 (36). The high expression of this marker was associated with International Federation of Gynaecology and Obstetrics (FIGO) grade, histological stage, and mortality (37). This study found that this indicator can also be used to assess the risk of tumor recurrence.

The maximum tumor diameter and para-aortic lymph node metastasis could also be independent risk factors for predicting the risk of tumor recurrence. A previous study suggested that lymph node dissection should be considered for all patients with the maximum diameter over 35 mm to prevent postoperative recurrence (38). In addition to being a risk factor for lymph node metastasis, tumor size is also a risk factor for cervical invasion, which is more likely to occur when the tumor diameter is larger than 3 cm (39). This study found that para-aortic lymph node metastases were associated with EC 5-year PFS, but not with pelvic lymph node metastases. Although it needs to be carefully verified, we speculate that lymph metastases at the first site may not significant affect the prognosis. A previous study found that, in endometrial cancer with stage IIIC disease, only when the second lymphatic station, like PALN is invaded it may indicate that the tumor has a strong invasive ability and a poor prognosis (40).

The whole tumor profile method was used to obtain all tumor information in this study, which is more accurate, though more time consuming than single-layer measurements. Previous studies have shown that the whole-tumor signatures outperformed single-slice signatures for prediction of LNM and advanced FIGO stage (41). Furthermore, we also used radiomics markers to predict postoperative pathological results such as DMI, FIGO, lymphatic metastasis and other established different models to indirectly evaluate the recurrence of tumor.

Based on the patients baseline clinical indicators and preoperative MR examination, the present study directly established a model to predict 5-PFS in EC patients, with the purpose of providing guidance for the clinical practice of endometrial cancer treatment and follow-up. Therefore, compared with other previous study, the present study was able to assess the tumor risk in the absence of preoperative pathological results. Based on the nomogram proposed in this study, we can calculate the patient score and make an accurate preoperative prediction of the 5-year recurrence and survival probability for each EC patient. In this way, it is more reasonable for clinicians to take more active treatment measures as soon as possible for patients suspected of having a higher risk of recurrence. Furthermore, more active follow-up should be carried out after surgery to detect recurrent lesions and intervene as soon as possible, to prolong and improve the quality of life. For patients with low risk, the rational application of this model can avoid overly aggressive surgical plans formulated by physicians, and also reduce the use of many unnecessary postoperative treatments (such as chemotherapy and radiotherapy). This provides a new strategy to avoiding pain, unnecessary economic loss and waste of medical resources caused by overtreatment.

This study has the following limitations that should be considered. First, this is a retrospective study and only includes those patients who had undergone surgery, which inevitably led to selective bias. Second, as a single centre study, whether the model proposed in this study is applicable to other MR systems remains unknown. Third, the sample size of this study is small, and the results need to be verified by large sample studies. In order to ensure the sample size for the training cohort and the accuracy of the model establishment, the samples have an unbalanced distribution. The small sample size of the test cohort may increase the uncertainty of its results. In addition, there are several extracted features, which may lead to the failure to include some indicators with strong correlation with PFS.

In conclusion, the radiomics model which incorporates clinical and cMR indicators was a good predictor of the relapse risk of EC. Using our radiomic parameter-based model and nomogram analysis can help guide preoperative non-invasive individualized evaluation for 5-year PFS and avoid possible under- or over-treatment.

## Data Availability Statement

The original contributions presented in the study are included in the article/**Supplementary Material**. Further inquiries can be directed to the corresponding author.

## Ethics Statement

The studies involving human participants were reviewed and approved by The Ethics Committee of the First Hospital of Qinhuangdao. Written informed consent for participation was not required for this study in accordance with the national legislation and the institutional requirements. Written informed consent was not obtained from the individual(s) for the publication of any potentially identifiable images or data included in this article.

## Author Contributions

DL, LY, and YC designed and coordinated the study, DL, DD, TZ, LL, ZW, JD, and YD carried out experiment and data process, and drafted the manuscript. All authors gave final approval for publication.

## Funding

This research was supported by National Natural Science Foundation of China (81871029) and Scientific Research Fund Project of Health Commission of Hebei Province (20200138).

## Conflict of Interest

The authors declare that the research was conducted in the absence of any commercial or financial relationships that could be construed as a potential conflict of interest.

## Publisher’s Note

All claims expressed in this article are solely those of the authors and do not necessarily represent those of their affiliated organizations, or those of the publisher, the editors and the reviewers. Any product that may be evaluated in this article, or claim that may be made by its manufacturer, is not guaranteed or endorsed by the publisher.
